# (Benzoato-κ^2^ *O*,*O*′)(5,5,7,12,12,14-hexa­methyl-1,4,8,11-tetra­azacyclo­tetra­decane-κ^4^ *N*,*N*′,*N*′′,*N*′′′)nickel(II) perchlorate monohydrate

**DOI:** 10.1107/S1600536808020564

**Published:** 2008-07-09

**Authors:** Guang-Chuan Ou, Min Zhang, Xian-You Yuan

**Affiliations:** a Department of Biology and Chemistry, Hunan University of Science and Engineering, Yongzhou Hunan 425100, People’s Republic of China

## Abstract

The Ni atom in the title salt, [Ni(C_7_H_5_O_2_)(C_16_H_36_N_4_)]ClO_4_·H_2_O, is in a six-coordinate octa­hedral geometry. The metal atom is chelated by the carboxyl­ate group, and the macrocyclic ligand adopts a folded configuration. The cation, anion and water mol­ecules engage in hydrogen bonding to form a layer structure.

## Related literature

For related literature, see: Jiang *et al.* (2005 ▶); Ou *et al.* (2008 ▶).
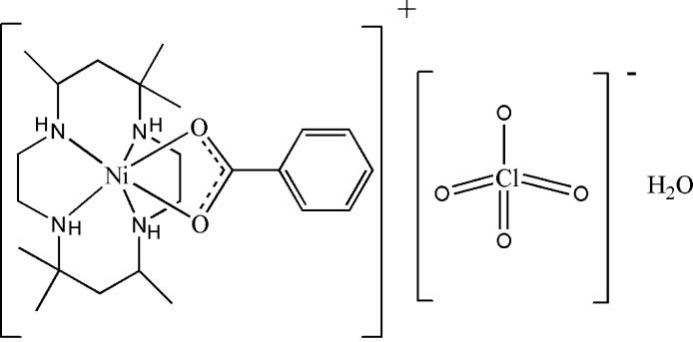


## Experimental

### 

#### Crystal data

[Ni(C_7_H_5_O_2_)(C_16_H_36_N_4_)]ClO_4_·H_2_O
*M* *_r_* = 581.77
Monoclinic, 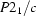

*a* = 15.1239 (14) Å
*b* = 8.9351 (8) Å
*c* = 20.9918 (19) Å
β = 102.414 (2)°
*V* = 2770.4 (4) Å^3^
*Z* = 4
Mo *K*α radiation
μ = 0.84 mm^−1^
*T* = 173 (2) K
0.48 × 0.40 × 0.21 mm

#### Data collection

Bruker SMART diffractometer
Absorption correction: multi-scan (*SADABS*; Sheldrick, 1996 ▶) *T*_min_ = 0.688, *T*_max_ = 0.843
15892 measured reflections
6007 independent reflections
4802 reflections with *I* > 2σ(*I*)
*R*_int_ = 0.023

#### Refinement

*R*[*F* ^2^ > 2σ(*F* ^2^)] = 0.035
*wR*(*F* ^2^) = 0.121
*S* = 1.10
6007 reflections
337 parameters
2 restraints
H atoms treated by a mixture of independent and constrained refinement
Δρ_max_ = 0.43 e Å^−3^
Δρ_min_ = −0.44 e Å^−3^

### 

Data collection: *SMART* (Bruker, 1999 ▶); cell refinement: *SAINT* (Bruker, 1999 ▶); data reduction: *SAINT*; program(s) used to solve structure: *SHELXS97* (Sheldrick, 2008 ▶); program(s) used to refine structure: *SHELXL97* (Sheldrick, 2008 ▶); molecular graphics: *SHELXTL* (Sheldrick, 2008 ▶); software used to prepare material for publication: *SHELXTL*.

## Supplementary Material

Crystal structure: contains datablocks I, global. DOI: 10.1107/S1600536808020564/ng2469sup1.cif

Structure factors: contains datablocks I. DOI: 10.1107/S1600536808020564/ng2469Isup2.hkl

Additional supplementary materials:  crystallographic information; 3D view; checkCIF report

## Figures and Tables

**Table:** 

Ni1—N4	2.0859 (19)
Ni1—N2	2.1053 (18)
Ni1—N3	2.117 (2)
Ni1—N1	2.1333 (19)
Ni1—O1	2.1379 (17)
Ni1—O2	2.1698 (16)

**Table:** 

O1—Ni1—O2	61.52 (6)

**Table 2:** Hydrogen-bond geometry (Å, °)

*D*—H⋯*A*	*D*—H	H⋯*A*	*D*⋯*A*	*D*—H⋯*A*
N3—H3*A*⋯O1*W*	0.93	2.16	3.080 (3)	168
O1*W*—H1*D*⋯O6	0.844 (19)	2.12 (3)	2.934 (4)	162 (6)
O1*W*—H1*E*⋯O2	0.86 (2)	2.18 (4)	2.931 (3)	146 (5)

**Fig. 1.**
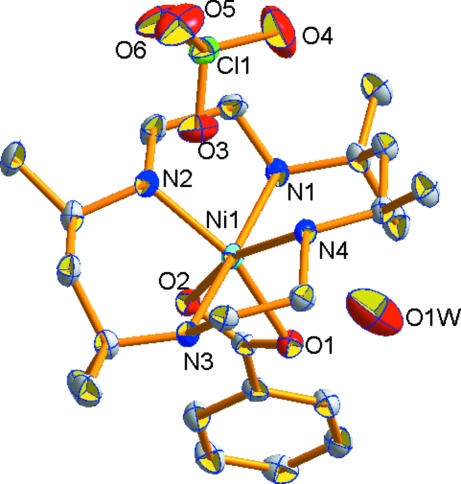
The molecular structure of (I), showing displacement ellipsoids at the 50% probability level.

**Fig. 2.**
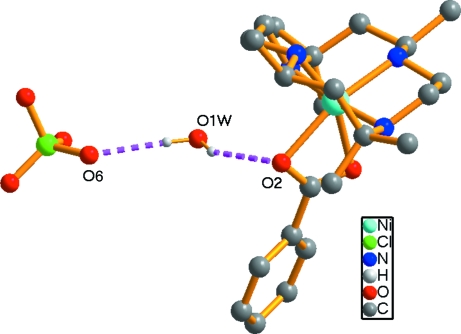
Two intermolecular hydrogen bond, O1w and O2 of benzoato anion, and O1w and O6 of [ClO4]- anion.

## supplementary crystallographic information

### Comment

It's important to control the geometries of *ML*^2+^ [*M* = Ni(II), Co(II), Cu(II)] with *cis*- or *trans*-conformation, since they form different structures and show different properties (Jiang *et al.*, 2005). A racemic nickel(II) complex with *cis*-conformation can be separated to two enantiomers by the reactions of [Ni(rac-*L*)]^2+^ with chiral amino acid such as phenylalanine (Ou *et al.*, 2008). Then we employ no chiral benzoic acid as separation reagent, but the result of experiment indicate a racemic complex of [Ni(rac-*L*)(bz)(ClO_4_)]H_2_O is obtained instead of two enantiomers. In the asymmetric unit of (I), contains one [Ni(rac-*L*)(bz)]^+^ cation, one [ClO_4_]^-^ anion and one water molecule. As illustrated in Fig.1, The six-coordinated Ni^2+^ of [Ni(rac-*L*)(bz)]^+^ cation display a distorted octahedral geometry by coordination with four N atoms of macrocyclic ligand *L* in a folded configuration, and two carboxylate oxygen atoms of benzoic acid in *cis*-position. The Ni—N distances ranging from 2.086 (19) to 2.133 (19) Å, are slight shorter than the Ni—O distance [2.138 (17) to 2.170 (16) Å] (Table 1). Neighbouring cations and anions are discrete, connected to each other through two intermolecular hydrogen bond (Table 2), water and oxygen atom of benzoato anion, and water and oxygen atom of [ClO_4_]^-^ anion (See Fig. 2).

### Experimental

benzoic acid (H_2_bz, 0.122 g, 1 mmol) was mixed with NaOH (0.040 g, 1 mmol) dissolved in 10 ml of water. To this solution was added [Ni(rac-*L*)](ClO_4_)_2_ (0.541 g, 1 mmol) dissolved in a minimum amount of CH_3_CN. The solution was left to stand at room temperature and blue crystals formed after several weeks(yield 53%).

### Refinement

H atoms attached to O (water) atoms were located in difference Fourier maps and condtrained to ride on their carrier atoms, with O—H distances in the range 0.82 Å, and with *U*_iso_ (H) = 1.5 times *U*_eq_ (O).

### Crystal data

**Table:**

[Ni(C_7_H_5_O_2_)(C_16_H_36_N_4_)]ClO_4_·H_2_O	*F*_000_ = 1240
*M**_r_* = 581.77	*D*_x_ = 1.395 Mg m^−^^3^
Monoclinic, *P*2_1_/*c*	Mo *K*α radiation λ = 0.71073 Å
Hall symbol: -P 2ybc	Cell parameters from 8118 reflections
*a* = 15.1239 (14) Å	θ = 2.7–27.1º
*b* = 8.9351 (8) Å	µ = 0.84 mm^−^^1^
*c* = 20.9918 (19) Å	*T* = 173 (2) K
β = 102.414 (2)º	Block, blue
*V* = 2770.4 (4) Å^3^	0.48 × 0.40 × 0.21 mm
*Z* = 4	

### Data collection

**Table:**

Bruker SMART diffractometer	6007 independent reflections
Radiation source: fine-focus sealed tube	4802 reflections with *I* > 2σ(*I*)
Monochromator: graphite	*R*_int_ = 0.023
*T* = 173(2) K	θ_max_ = 27.1º
φ and ω scans	θ_min_ = 1.4º
Absorption correction: multi-scan(SADABS; Sheldrick, 1996)	*h* = −16→19
*T*_min_ = 0.688, *T*_max_ = 0.843	*k* = −11→11
15892 measured reflections	*l* = −26→24

### Refinement

**Table:**

Refinement on *F*^2^	Secondary atom site location: difference Fourier map
Least-squares matrix: full	Hydrogen site location: inferred from neighbouring sites
*R*[*F*^2^ > 2σ(*F*^2^)] = 0.036	H atoms treated by a mixture of independent and constrained refinement
*wR*(*F*^2^) = 0.121	*w* = 1/[σ^2^(*F*_o_^2^) + (0.0673*P*)^2^ + 1.378*P*] where *P* = (*F*_o_^2^ + 2*F*_c_^2^)/3
*S* = 1.10	(Δ/σ)_max_ < 0.001
6007 reflections	Δρ_max_ = 0.43 e Å^−^^3^
337 parameters	Δρ_min_ = −0.44 e Å^−^^3^
2 restraints	Extinction correction: none
Primary atom site location: structure-invariant direct methods	

### Special details

**Table:**

Geometry. All e.s.d.'s (except the e.s.d. in the dihedral angle between two l.s. planes) are estimated using the full covariance matrix. The cell e.s.d.'s are taken into account individually in the estimation of e.s.d.'s in distances, angles and torsion angles; correlations between e.s.d.'s in cell parameters are only used when they are defined by crystal symmetry. An approximate (isotropic) treatment of cell e.s.d.'s is used for estimating e.s.d.'s involving l.s. planes.
Refinement. Refinement of *F*^2^ against ALL reflections. The weighted *R*-factor *wR* and goodness of fit *S* are based on *F*^2^, conventional *R*-factors *R* are based on *F*, with *F* set to zero for negative *F*^2^. The threshold expression of *F*^2^ > σ(*F*^2^) is used only for calculating *R*-factors(gt) *etc*. and is not relevant to the choice of reflections for refinement. *R*-factors based on *F*^2^ are statistically about twice as large as those based on *F*, and *R*- factors based on ALL data will be even larger.

### Fractional atomic coordinates and isotropic or equivalent isotropic displacement parameters (Å^2^)

**Table:**

	*x*	*y*	*z*	*U*_iso_*/*U*_eq_	
Ni1	0.250097 (18)	0.59158 (3)	0.132452 (13)	0.01980 (10)	
N4	0.15871 (13)	0.4136 (2)	0.11775 (9)	0.0229 (4)	
H4D	0.1685	0.3575	0.1559	0.027*	
O1	0.30893 (11)	0.79837 (19)	0.11139 (8)	0.0256 (4)	
O2	0.20094 (11)	0.69355 (18)	0.03764 (8)	0.0256 (4)	
N1	0.16886 (12)	0.7161 (2)	0.18507 (9)	0.0226 (4)	
H1C	0.1839	0.8159	0.1806	0.027*	
N3	0.33157 (13)	0.4525 (2)	0.08693 (9)	0.0237 (4)	
H3A	0.3228	0.4868	0.0442	0.028*	
N2	0.33164 (12)	0.5457 (2)	0.22499 (9)	0.0209 (4)	
H2C	0.3111	0.4562	0.2392	0.025*	
C9	0.20586 (16)	0.6787 (3)	0.25445 (11)	0.0259 (5)	
H9A	0.1799	0.5828	0.2653	0.031*	
H9B	0.1890	0.7577	0.2827	0.031*	
C18	0.27474 (16)	0.9066 (3)	0.00497 (11)	0.0233 (5)	
C17	0.26017 (15)	0.7949 (3)	0.05489 (11)	0.0232 (5)	
C10	0.30721 (16)	0.6661 (3)	0.26643 (12)	0.0277 (5)	
H10A	0.3333	0.7623	0.2560	0.033*	
H10B	0.3321	0.6431	0.3130	0.033*	
C13	0.45580 (16)	0.4159 (3)	0.18539 (12)	0.0273 (5)	
H13A	0.4254	0.3212	0.1924	0.033*	
H13B	0.5218	0.3976	0.1982	0.033*	
C11	0.43209 (15)	0.5309 (3)	0.23209 (11)	0.0249 (5)	
H11	0.4566	0.6300	0.2221	0.030*	
C2	0.18611 (17)	0.3218 (3)	0.06704 (12)	0.0296 (5)	
H2A	0.1664	0.3708	0.0240	0.036*	
H2B	0.1566	0.2225	0.0650	0.036*	
C14	0.43295 (16)	0.4485 (3)	0.11189 (12)	0.0273 (5)	
C16	0.47053 (18)	0.5992 (3)	0.09632 (14)	0.0355 (6)	
H16A	0.5365	0.5997	0.1117	0.053*	
H16B	0.4439	0.6791	0.1182	0.053*	
H16C	0.4553	0.6158	0.0491	0.053*	
C3	0.06071 (15)	0.4551 (3)	0.10069 (12)	0.0273 (5)	
H3	0.0499	0.5183	0.0604	0.033*	
C8	0.02322 (18)	0.7801 (3)	0.21821 (14)	0.0375 (6)	
H8A	0.0347	0.7164	0.2571	0.056*	
H8B	−0.0422	0.7892	0.2014	0.056*	
H8C	0.0491	0.8796	0.2296	0.056*	
C5	0.03588 (16)	0.5467 (3)	0.15609 (13)	0.0300 (5)	
H5A	−0.0309	0.5462	0.1496	0.036*	
H5B	0.0600	0.4931	0.1974	0.036*	
C21	0.3060 (2)	1.1047 (3)	−0.08971 (13)	0.0344 (6)	
H21	0.3168	1.1726	−0.1220	0.041*	
C23	0.20363 (17)	0.9519 (3)	−0.04531 (13)	0.0318 (6)	
H23	0.1443	0.9144	−0.0475	0.038*	
C22	0.21979 (19)	1.0518 (3)	−0.09210 (13)	0.0368 (6)	
H22	0.1712	1.0839	−0.1259	0.044*	
C20	0.37688 (18)	1.0589 (3)	−0.04021 (13)	0.0313 (6)	
H20	0.4365	1.0940	−0.0391	0.038*	
C1	0.28810 (17)	0.3027 (3)	0.08246 (13)	0.0312 (6)	
H1A	0.3076	0.2488	0.1243	0.037*	
H1B	0.3066	0.2432	0.0478	0.037*	
C7	0.03832 (18)	0.8008 (3)	0.10272 (13)	0.0362 (6)	
H7A	0.0533	0.9065	0.1118	0.054*	
H7B	−0.0271	0.7904	0.0863	0.054*	
H7C	0.0703	0.7638	0.0699	0.054*	
C19	0.36099 (16)	0.9624 (3)	0.00744 (12)	0.0278 (5)	
H19	0.4094	0.9340	0.0422	0.033*	
C12	0.47882 (18)	0.4865 (3)	0.30144 (12)	0.0351 (6)	
H12A	0.4629	0.5584	0.3324	0.053*	
H12B	0.5446	0.4866	0.3054	0.053*	
H12C	0.4590	0.3862	0.3110	0.053*	
C4	−0.00056 (18)	0.3171 (3)	0.08730 (14)	0.0389 (7)	
H4A	0.0125	0.2620	0.0500	0.058*	
H4B	−0.0641	0.3488	0.0774	0.058*	
H4C	0.0107	0.2523	0.1259	0.058*	
C6	0.06727 (15)	0.7097 (3)	0.16554 (11)	0.0266 (5)	
C15	0.47577 (19)	0.3267 (3)	0.07671 (13)	0.0375 (6)	
H15A	0.5419	0.3357	0.0885	0.056*	
H15B	0.4550	0.3386	0.0294	0.056*	
H15C	0.4578	0.2279	0.0897	0.056*	
O1W	0.2792 (2)	0.5335 (4)	−0.05895 (13)	0.0872 (11)	
H1D	0.246 (3)	0.505 (7)	−0.0943 (18)	0.131*	
H1E	0.238 (3)	0.581 (6)	−0.045 (3)	0.131*	
Cl1	0.23445 (4)	0.37222 (7)	−0.24307 (3)	0.03201 (16)	
O3	0.21061 (14)	0.2166 (2)	−0.24210 (9)	0.0415 (5)	
O5	0.19455 (19)	0.4317 (2)	−0.30582 (11)	0.0585 (7)	
O4	0.32947 (18)	0.3837 (4)	−0.23043 (17)	0.0865 (10)	
O6	0.1992 (2)	0.4479 (3)	−0.19397 (11)	0.0654 (7)	

### Atomic displacement parameters (Å^2^)

**Table:**

	*U*^11^	*U*^22^	*U*^33^	*U*^12^	*U*^13^	*U*^23^
Ni1	0.01889 (16)	0.02163 (17)	0.01882 (16)	−0.00185 (11)	0.00392 (11)	0.00090 (11)
N4	0.0240 (10)	0.0251 (10)	0.0189 (9)	−0.0024 (8)	0.0033 (8)	0.0007 (7)
O1	0.0255 (8)	0.0277 (9)	0.0232 (8)	−0.0025 (7)	0.0043 (7)	0.0029 (7)
O2	0.0244 (8)	0.0258 (9)	0.0255 (8)	−0.0029 (7)	0.0032 (7)	0.0017 (7)
N1	0.0207 (9)	0.0227 (10)	0.0246 (10)	−0.0001 (8)	0.0057 (8)	0.0002 (8)
N3	0.0209 (9)	0.0282 (11)	0.0224 (10)	−0.0001 (8)	0.0056 (8)	0.0008 (8)
N2	0.0192 (9)	0.0232 (10)	0.0198 (9)	−0.0005 (7)	0.0034 (7)	0.0013 (7)
C9	0.0291 (12)	0.0268 (13)	0.0226 (11)	0.0015 (10)	0.0074 (9)	−0.0020 (9)
C18	0.0246 (11)	0.0233 (12)	0.0235 (11)	0.0021 (9)	0.0083 (9)	0.0006 (9)
C17	0.0201 (11)	0.0244 (12)	0.0256 (11)	0.0035 (9)	0.0063 (9)	0.0003 (9)
C10	0.0284 (13)	0.0283 (13)	0.0245 (12)	0.0003 (10)	0.0012 (10)	−0.0041 (10)
C13	0.0232 (12)	0.0300 (13)	0.0276 (12)	0.0031 (9)	0.0034 (10)	0.0012 (10)
C11	0.0208 (11)	0.0282 (13)	0.0249 (12)	−0.0010 (9)	0.0028 (9)	0.0030 (9)
C2	0.0296 (13)	0.0339 (14)	0.0257 (12)	−0.0074 (10)	0.0067 (10)	−0.0093 (10)
C14	0.0221 (12)	0.0329 (13)	0.0282 (12)	0.0024 (10)	0.0082 (10)	0.0010 (10)
C16	0.0263 (13)	0.0443 (17)	0.0367 (15)	−0.0027 (11)	0.0090 (11)	0.0089 (12)
C3	0.0193 (11)	0.0340 (14)	0.0269 (12)	−0.0038 (10)	0.0015 (9)	−0.0004 (10)
C8	0.0302 (13)	0.0434 (17)	0.0420 (16)	0.0040 (12)	0.0142 (12)	−0.0067 (12)
C5	0.0205 (12)	0.0370 (14)	0.0331 (13)	−0.0047 (10)	0.0073 (10)	−0.0024 (11)
C21	0.0448 (16)	0.0312 (14)	0.0316 (14)	0.0048 (11)	0.0182 (12)	0.0095 (11)
C23	0.0252 (12)	0.0350 (14)	0.0343 (14)	−0.0005 (10)	0.0042 (10)	0.0050 (11)
C22	0.0345 (14)	0.0425 (16)	0.0311 (14)	0.0068 (12)	0.0018 (11)	0.0102 (12)
C20	0.0285 (13)	0.0333 (14)	0.0353 (14)	−0.0006 (11)	0.0138 (11)	0.0035 (11)
C1	0.0333 (14)	0.0250 (13)	0.0377 (14)	−0.0004 (10)	0.0129 (11)	−0.0067 (10)
C7	0.0269 (13)	0.0417 (16)	0.0378 (14)	0.0092 (11)	0.0023 (11)	0.0027 (12)
C19	0.0234 (12)	0.0320 (13)	0.0284 (12)	0.0027 (10)	0.0066 (10)	0.0046 (10)
C12	0.0290 (13)	0.0463 (17)	0.0266 (13)	0.0064 (12)	−0.0017 (10)	0.0001 (12)
C4	0.0270 (13)	0.0446 (17)	0.0444 (16)	−0.0144 (12)	0.0060 (11)	−0.0119 (13)
C6	0.0212 (11)	0.0329 (14)	0.0253 (12)	0.0016 (10)	0.0045 (9)	−0.0017 (10)
C15	0.0332 (14)	0.0447 (17)	0.0371 (15)	0.0087 (12)	0.0134 (11)	0.0004 (12)
O1W	0.108 (3)	0.104 (2)	0.0420 (15)	0.051 (2)	−0.0002 (15)	−0.0162 (15)
Cl1	0.0377 (3)	0.0263 (3)	0.0303 (3)	0.0022 (2)	0.0035 (3)	−0.0017 (2)
O3	0.0604 (13)	0.0272 (10)	0.0369 (10)	−0.0006 (9)	0.0105 (9)	0.0005 (8)
O5	0.096 (2)	0.0360 (12)	0.0376 (12)	0.0114 (12)	0.0018 (12)	0.0082 (9)
O4	0.0395 (14)	0.112 (3)	0.104 (2)	−0.0220 (15)	0.0060 (15)	0.0054 (19)
O6	0.097 (2)	0.0534 (15)	0.0436 (13)	0.0220 (14)	0.0098 (13)	−0.0208 (11)

### Geometric parameters (Å, °)

**Table:**

Ni1—N4	2.0859 (19)	C16—H16C	0.9800
Ni1—N2	2.1053 (18)	C3—C4	1.532 (3)
Ni1—N3	2.117 (2)	C3—C5	1.533 (4)
Ni1—N1	2.1333 (19)	C3—H3	1.0000
Ni1—O1	2.1379 (17)	C8—C6	1.542 (3)
Ni1—O2	2.1698 (16)	C8—H8A	0.9800
N4—C2	1.472 (3)	C8—H8B	0.9800
N4—C3	1.495 (3)	C8—H8C	0.9800
N4—H4D	0.9300	C5—C6	1.531 (4)
O1—C17	1.255 (3)	C5—H5A	0.9900
O2—C17	1.271 (3)	C5—H5B	0.9900
N1—C9	1.482 (3)	C21—C22	1.377 (4)
N1—C6	1.504 (3)	C21—C20	1.385 (4)
N1—H1C	0.9300	C21—H21	0.9500
N3—C1	1.485 (3)	C23—C22	1.387 (4)
N3—C14	1.510 (3)	C23—H23	0.9500
N3—H3A	0.9300	C22—H22	0.9500
N2—C10	1.480 (3)	C20—C19	1.380 (3)
N2—C11	1.500 (3)	C20—H20	0.9500
N2—H2C	0.9300	C1—H1A	0.9900
C9—C10	1.503 (3)	C1—H1B	0.9900
C9—H9A	0.9900	C7—C6	1.531 (4)
C9—H9B	0.9900	C7—H7A	0.9800
C18—C19	1.387 (3)	C7—H7B	0.9800
C18—C23	1.395 (3)	C7—H7C	0.9800
C18—C17	1.497 (3)	C19—H19	0.9500
C10—H10A	0.9900	C12—H12A	0.9800
C10—H10B	0.9900	C12—H12B	0.9800
C13—C11	1.515 (3)	C12—H12C	0.9800
C13—C14	1.535 (3)	C4—H4A	0.9800
C13—H13A	0.9900	C4—H4B	0.9800
C13—H13B	0.9900	C4—H4C	0.9800
C11—C12	1.528 (3)	C15—H15A	0.9800
C11—H11	1.0000	C15—H15B	0.9800
C2—C1	1.516 (3)	C15—H15C	0.9800
C2—H2A	0.9900	O1W—H1D	0.844 (19)
C2—H2B	0.9900	O1W—H1E	0.86 (2)
C14—C16	1.524 (4)	Cl1—O4	1.408 (3)
C14—C15	1.535 (4)	Cl1—O5	1.428 (2)
C16—H16A	0.9800	Cl1—O6	1.428 (2)
C16—H16B	0.9800	Cl1—O3	1.437 (2)
			
N4—Ni1—N2	103.07 (8)	C16—C14—C15	108.0 (2)
N4—Ni1—N3	85.25 (8)	C13—C14—C15	108.8 (2)
N2—Ni1—N3	91.14 (7)	C14—C16—H16A	109.5
N4—Ni1—N1	92.13 (8)	C14—C16—H16B	109.5
N2—Ni1—N1	84.96 (7)	H16A—C16—H16B	109.5
N3—Ni1—N1	174.71 (8)	C14—C16—H16C	109.5
N4—Ni1—O1	156.97 (7)	H16A—C16—H16C	109.5
N2—Ni1—O1	99.89 (7)	H16B—C16—H16C	109.5
N3—Ni1—O1	96.05 (7)	N4—C3—C4	111.9 (2)
N1—Ni1—O1	88.16 (7)	N4—C3—C5	110.04 (19)
N4—Ni1—O2	95.68 (7)	C4—C3—C5	109.4 (2)
N2—Ni1—O2	160.97 (7)	N4—C3—H3	108.5
N3—Ni1—O2	87.15 (7)	C4—C3—H3	108.5
N1—Ni1—O2	97.70 (7)	C5—C3—H3	108.5
O1—Ni1—O2	61.52 (6)	C6—C8—H8A	109.5
C2—N4—C3	112.56 (18)	C6—C8—H8B	109.5
C2—N4—Ni1	104.54 (14)	H8A—C8—H8B	109.5
C3—N4—Ni1	115.95 (15)	C6—C8—H8C	109.5
C2—N4—H4D	107.8	H8A—C8—H8C	109.5
C3—N4—H4D	107.8	H8B—C8—H8C	109.5
Ni1—N4—H4D	107.8	C6—C5—C3	119.1 (2)
C17—O1—Ni1	89.32 (14)	C6—C5—H5A	107.5
C17—O2—Ni1	87.50 (13)	C3—C5—H5A	107.5
C9—N1—C6	114.04 (17)	C6—C5—H5B	107.5
C9—N1—Ni1	104.68 (13)	C3—C5—H5B	107.5
C6—N1—Ni1	120.50 (14)	H5A—C5—H5B	107.0
C9—N1—H1C	105.5	C22—C21—C20	120.0 (2)
C6—N1—H1C	105.5	C22—C21—H21	120.0
Ni1—N1—H1C	105.5	C20—C21—H21	120.0
C1—N3—C14	113.78 (19)	C22—C23—C18	119.9 (2)
C1—N3—Ni1	105.34 (14)	C22—C23—H23	120.1
C14—N3—Ni1	120.21 (15)	C18—C23—H23	120.1
C1—N3—H3A	105.4	C21—C22—C23	120.3 (2)
C14—N3—H3A	105.4	C21—C22—H22	119.9
Ni1—N3—H3A	105.4	C23—C22—H22	119.9
C10—N2—C11	112.42 (18)	C19—C20—C21	120.0 (2)
C10—N2—Ni1	103.31 (14)	C19—C20—H20	120.0
C11—N2—Ni1	119.26 (14)	C21—C20—H20	120.0
C10—N2—H2C	107.1	N3—C1—C2	109.2 (2)
C11—N2—H2C	107.1	N3—C1—H1A	109.8
Ni1—N2—H2C	107.1	C2—C1—H1A	109.8
N1—C9—C10	109.72 (18)	N3—C1—H1B	109.8
N1—C9—H9A	109.7	C2—C1—H1B	109.8
C10—C9—H9A	109.7	H1A—C1—H1B	108.3
N1—C9—H9B	109.7	C6—C7—H7A	109.5
C10—C9—H9B	109.7	C6—C7—H7B	109.5
H9A—C9—H9B	108.2	H7A—C7—H7B	109.5
C19—C18—C23	119.3 (2)	C6—C7—H7C	109.5
C19—C18—C17	119.5 (2)	H7A—C7—H7C	109.5
C23—C18—C17	121.1 (2)	H7B—C7—H7C	109.5
O1—C17—O2	121.4 (2)	C20—C19—C18	120.4 (2)
O1—C17—C18	120.0 (2)	C20—C19—H19	119.8
O2—C17—C18	118.5 (2)	C18—C19—H19	119.8
O1—C17—Ni1	60.09 (12)	C11—C12—H12A	109.5
O2—C17—Ni1	61.52 (12)	C11—C12—H12B	109.5
C18—C17—Ni1	172.92 (16)	H12A—C12—H12B	109.5
N2—C10—C9	109.31 (19)	C11—C12—H12C	109.5
N2—C10—H10A	109.8	H12A—C12—H12C	109.5
C9—C10—H10A	109.8	H12B—C12—H12C	109.5
N2—C10—H10B	109.8	C3—C4—H4A	109.5
C9—C10—H10B	109.8	C3—C4—H4B	109.5
H10A—C10—H10B	108.3	H4A—C4—H4B	109.5
C11—C13—C14	119.2 (2)	C3—C4—H4C	109.5
C11—C13—H13A	107.5	H4A—C4—H4C	109.5
C14—C13—H13A	107.5	H4B—C4—H4C	109.5
C11—C13—H13B	107.5	N1—C6—C7	107.51 (19)
C14—C13—H13B	107.5	N1—C6—C5	109.96 (19)
H13A—C13—H13B	107.0	C7—C6—C5	111.7 (2)
N2—C11—C13	111.78 (19)	N1—C6—C8	111.26 (19)
N2—C11—C12	111.64 (19)	C7—C6—C8	108.2 (2)
C13—C11—C12	108.4 (2)	C5—C6—C8	108.1 (2)
N2—C11—H11	108.3	C14—C15—H15A	109.5
C13—C11—H11	108.3	C14—C15—H15B	109.5
C12—C11—H11	108.3	H15A—C15—H15B	109.5
N4—C2—C1	109.87 (19)	C14—C15—H15C	109.5
N4—C2—H2A	109.7	H15A—C15—H15C	109.5
C1—C2—H2A	109.7	H15B—C15—H15C	109.5
N4—C2—H2B	109.7	H1D—O1W—H1E	96 (5)
C1—C2—H2B	109.7	O4—Cl1—O5	110.99 (19)
H2A—C2—H2B	108.2	O4—Cl1—O6	110.70 (19)
N3—C14—C16	107.6 (2)	O5—Cl1—O6	109.89 (15)
N3—C14—C13	110.23 (19)	O4—Cl1—O3	108.47 (17)
C16—C14—C13	111.7 (2)	O5—Cl1—O3	108.38 (13)
N3—C14—C15	110.6 (2)	O6—Cl1—O3	108.32 (15)
			
N2—Ni1—N4—C2	−108.67 (15)	C19—C18—C17—O2	−145.7 (2)
N3—Ni1—N4—C2	−18.60 (15)	C23—C18—C17—O2	32.2 (3)
N1—Ni1—N4—C2	166.01 (15)	N4—Ni1—C17—O1	174.52 (12)
O1—Ni1—N4—C2	75.7 (2)	N2—Ni1—C17—O1	−9.01 (17)
O2—Ni1—N4—C2	68.05 (15)	N3—Ni1—C17—O1	−100.98 (13)
C17—Ni1—N4—C2	68.56 (17)	N1—Ni1—C17—O1	78.34 (14)
N2—Ni1—N4—C3	126.78 (15)	O2—Ni1—C17—O1	175.5 (2)
N3—Ni1—N4—C3	−143.15 (16)	N4—Ni1—C17—O2	−0.99 (17)
N1—Ni1—N4—C3	41.46 (16)	N2—Ni1—C17—O2	175.48 (12)
O1—Ni1—N4—C3	−48.8 (3)	N3—Ni1—C17—O2	83.51 (13)
O2—Ni1—N4—C3	−56.50 (16)	N1—Ni1—C17—O2	−97.16 (13)
C17—Ni1—N4—C3	−55.99 (18)	O1—Ni1—C17—O2	−175.5 (2)
N4—Ni1—O1—C17	−11.3 (3)	C11—N2—C10—C9	177.47 (19)
N2—Ni1—O1—C17	173.02 (13)	Ni1—N2—C10—C9	47.6 (2)
N3—Ni1—O1—C17	80.80 (14)	N1—C9—C10—N2	−60.6 (3)
N1—Ni1—O1—C17	−102.42 (14)	C10—N2—C11—C13	−175.36 (19)
O2—Ni1—O1—C17	−2.63 (13)	Ni1—N2—C11—C13	−54.3 (2)
N4—Ni1—O2—C17	179.21 (13)	C10—N2—C11—C12	63.1 (3)
N2—Ni1—O2—C17	−10.6 (3)	Ni1—N2—C11—C12	−175.85 (17)
N3—Ni1—O2—C17	−95.85 (14)	C14—C13—C11—N2	68.1 (3)
N1—Ni1—O2—C17	86.27 (14)	C14—C13—C11—C12	−168.4 (2)
O1—Ni1—O2—C17	2.60 (12)	C3—N4—C2—C1	171.8 (2)
N4—Ni1—N1—C9	93.70 (15)	Ni1—N4—C2—C1	45.1 (2)
N2—Ni1—N1—C9	−9.24 (14)	C1—N3—C14—C16	164.6 (2)
O1—Ni1—N1—C9	−109.34 (14)	Ni1—N3—C14—C16	−69.1 (2)
O2—Ni1—N1—C9	−170.27 (14)	C1—N3—C14—C13	−73.3 (2)
C17—Ni1—N1—C9	−139.25 (14)	Ni1—N3—C14—C13	52.9 (2)
N4—Ni1—N1—C6	−36.32 (17)	C1—N3—C14—C15	47.0 (3)
N2—Ni1—N1—C6	−139.26 (17)	Ni1—N3—C14—C15	173.21 (16)
O1—Ni1—N1—C6	120.64 (17)	C11—C13—C14—N3	−66.7 (3)
O2—Ni1—N1—C6	59.71 (17)	C11—C13—C14—C16	52.9 (3)
C17—Ni1—N1—C6	90.73 (17)	C11—C13—C14—C15	171.9 (2)
N4—Ni1—N3—C1	−10.58 (15)	C2—N4—C3—C4	56.6 (3)
N2—Ni1—N3—C1	92.45 (15)	Ni1—N4—C3—C4	176.92 (17)
O1—Ni1—N3—C1	−167.48 (15)	C2—N4—C3—C5	178.5 (2)
O2—Ni1—N3—C1	−106.52 (15)	Ni1—N4—C3—C5	−61.2 (2)
C17—Ni1—N3—C1	−137.33 (15)	N4—C3—C5—C6	74.1 (3)
N4—Ni1—N3—C14	−140.64 (17)	C4—C3—C5—C6	−162.6 (2)
N2—Ni1—N3—C14	−37.62 (17)	C19—C18—C23—C22	0.0 (4)
O1—Ni1—N3—C14	62.45 (17)	C17—C18—C23—C22	−177.9 (2)
O2—Ni1—N3—C14	123.41 (17)	C20—C21—C22—C23	−0.4 (4)
N4—Ni1—N2—C10	−111.54 (15)	C18—C23—C22—C21	1.0 (4)
N3—Ni1—N2—C10	163.07 (15)	C22—C21—C20—C19	−1.2 (4)
N1—Ni1—N2—C10	−20.52 (14)	C14—N3—C1—C2	171.41 (19)
O1—Ni1—N2—C10	66.73 (15)	Ni1—N3—C1—C2	37.7 (2)
O2—Ni1—N2—C10	78.5 (3)	N4—C2—C1—N3	−58.1 (3)
C17—Ni1—N2—C10	71.37 (17)	C21—C20—C19—C18	2.2 (4)
N4—Ni1—N2—C11	122.90 (17)	C23—C18—C19—C20	−1.6 (4)
N3—Ni1—N2—C11	37.51 (17)	C17—C18—C19—C20	176.3 (2)
N1—Ni1—N2—C11	−146.08 (17)	C9—N1—C6—C7	160.6 (2)
O1—Ni1—N2—C11	−58.84 (17)	Ni1—N1—C6—C7	−73.6 (2)
O2—Ni1—N2—C11	−47.1 (3)	C9—N1—C6—C5	−77.6 (2)
C6—N1—C9—C10	171.69 (19)	Ni1—N1—C6—C5	48.2 (2)
Ni1—N1—C9—C10	38.0 (2)	C9—N1—C6—C8	42.2 (3)
Ni1—O1—C17—O2	4.6 (2)	Ni1—N1—C6—C8	168.01 (17)
Ni1—O1—C17—C18	−171.82 (19)	C3—C5—C6—N1	−65.4 (3)
Ni1—O2—C17—O1	−4.6 (2)	C3—C5—C6—C7	53.9 (3)
C19—C18—C17—O1	30.9 (3)	C3—C5—C6—C8	172.9 (2)
C23—C18—C17—O1	−151.3 (2)		

### Hydrogen-bond geometry (Å, °)

**Table:**

*D*—H···*A*	*D*—H	H···*A*	*D*···*A*	*D*—H···*A*
N3—H3A···O1W	0.93	2.16	3.080 (3)	168
O1W—H1D···O6	0.844 (19)	2.12 (3)	2.934 (4)	162 (6)
O1W—H1E···O2	0.86 (2)	2.18 (4)	2.931 (3)	146 (5)
